# Male Lineages in Brazil: Intercontinental Admixture and Stratification of the European Background

**DOI:** 10.1371/journal.pone.0152573

**Published:** 2016-04-05

**Authors:** Rafael Resque, Leonor Gusmão, Maria Geppert, Lutz Roewer, Teresinha Palha, Luis Alvarez, Ândrea Ribeiro-dos-Santos, Sidney Santos

**Affiliations:** 1 Laboratório de Genética Humana e Médica, Instituto de Ciências Biológicas, Universidade Federal do Pará, Belém, Brazil; 2 DNA Diagnostic Laboratory (LDD), Institute of Biology, State University of Rio de Janeiro (UERJ), Rio de Janeiro, Brazil; 3 IPATIMUP—Institute of Molecular Pathology and Immunology of the University of Porto, Porto, Portugal; 4 Instituto de Investigação e Inovação em Saúde, Universidade do Porto, Porto, Portugal; 5 Department of Forensic Genetics, Institute of Legal Medicine and Forensic Sciences, Charité—Universitätsmedizin Berlin, Berlin, Germany; 6 Laboratório de Toxicologia e Química Farmacêutica, Departamento de Ciências da Saúde e Biológicas, Universidade Federal do Amapá, Macapá, Brazil; 7 Laboratório de Genética Forense, Instituto de Criminalística, Centro de Perícias Científicas Renato Chaves, Belém, Pará, Brasil; 8 Núcleo de Pesquisas em Oncologia, Universidade Federal do Pará, Belém, Brazil; Universitat Pompeu Fabra, SPAIN

## Abstract

The non-recombining nature of the Y chromosome and the well-established phylogeny of Y-specific Single Nucleotide Polymorphisms (Y-SNPs) make them useful for defining haplogroups with high geographical specificity; therefore, they are more apt than the Y-STRs to detect population stratification in admixed populations from diverse continental origins. Different Y-SNP typing strategies have been described to address issues of population history and movements within geographic territories of interest. In this study, we investigated a set of 41 Y-SNPs in 1217 unrelated males from the five Brazilian geopolitical regions, aiming to disclose the genetic structure of male lineages in the country. A population comparison based on pairwise *F*_*ST*_ genetic distances did not reveal statistically significant differences in haplogroup frequency distributions among populations from the different regions. The genetic differences observed among regions were, however, consistent with the colonization history of the country. The sample from the Northern region presented the highest Native American ancestry (8.4%), whereas the more pronounced African contribution could be observed in the Northeastern population (15.1%). The Central-Western and Southern samples showed the higher European contributions (95.7% and 93.6%, respectively). The Southeastern region presented significant European (86.1%) and African (12.0%) contributions. The subtyping of the most frequent European lineage in Brazil (R1b1a-M269) allowed differences in the genetic European background of the five Brazilian regions to be investigated for the first time.

## Introduction

Due to the way it was formed, the Brazilian population exhibits some peculiar characteristics. In the early sixteenth century, Brazil came under the influence of three main groups: the Native Americans who already inhabited the region at the time; the Portuguese, who arrived in the territory in the mid-1500s, and the Africans that were brought by the Portuguese during the slave trade period. By this time, French and Dutch settlers also arrived in an attempt to colonize the region, but they were soon expelled by the Portuguese. From 1808 on, other people migrated to the country, including Spaniards, Italians, Germans, Syrians, Lebanese and Japanese, also contributing to the formation of the current population [1–5].

Brazil is a country of continental extension, and it is currently divided into five main geopolitical regions (North, Northeast, Central-West, Southeast and South) with diverse histories of colonization and settlement, a fact that is reflected in the genetic structure of the current Brazilian population [5–8].

The existing heterogeneity among Brazilian populations was mainly shaped by differential maternal heritage in a biased mating environment. Indeed, the early European colonization involved mainly sailors, soldiers, deportees and wood traders, and the migration of European women was insignificant, which favored mating between European men and indigenous or African women [2,4].

When using genetic markers with low mutation rates and different inheritance patterns, a clear heterogeneity can be observed in the genetic contribution of each parental population to the different geopolitical regions of the country as a result of diverse colonization histories [6–8]. However, the ability to detect heterogeneity considerably decreases when markers with high mutation rates are investigated because this type of polymorphism is much more prone to detecting differentiation within rather than between populations [9–11].

Due to the high mutation rate of the Y-STRs and the predominant European male contribution to the Brazilian population, most studies based on the analysis of Y-STRs have failed to detect statistically significant differences between admixed populations in the country [9–11]. In the largest study performed until now on the Y-STR haplotype distribution in Brazilian populations, Palha et al. [10] also failed to detect the substructure for 23 Y-STR loci in more than 2,000 Y chromosomes in 17 different admixed populations from the five regions of the country. However, by enlarging the size of the sample representing the South West region of the country, Oliveira et al. [11] could detect a significant difference between this region and the North, for a smaller set of 17 Y-STRs, which was further supported by the results of the Y-SNP analysis.

Despite having a lower intra-population discrimination power than Y-STRs, Y-SNPs are more powerful in revealing the differences among populations characterized by diverse levels of admixture. The non-recombining nature of the Y chromosome associated with a high geographical specificity of the haplogroups formed by sets of SNPs distributed along the chromosome make the Y-SNPs the most appropriate genetic markers to detect population stratification of male lineages in admixed populations [11].

Until now, a limited number of studies using Y-SNPs have been performed to characterize admixed populations in Brazil, and they have focused on small population groups, reporting only estimates of the portions of Native American, European, and African contributions to the current populations [11–19]. These studies show greater European contribution in admixed populations from urban centers along with reduced African and Native American contributions, which can be more or less important depending on the region [11–15]. However, in specific population groups such as Afro-descendant communities and Native American isolated tribes, the more prevalent male contributions are from African and American origin, respectively [16–19].

Nevertheless, no attempts have been made to detect the differences concerning the different European sources of the male lineages currently existing in Brazil, by increasing the resolution of the main European haplogroups.

Therefore, to better characterize the male lineage background of Brazil, in the present work a large set of highly informative Y-SNPs was for the first time investigated in representative samples from all Brazilian geopolitical regions. Moreover, to discriminate contributions from different countries in Europe, a group of Y-SNPs was selected to increase the resolution inside haplogroup R1b1a-M269, the main representative of the European male lineages present in Brazil. In this way, we were able to not only determine the different continental source of the Y chromosomes present in Brazilian admixed populations belonging to the five Brazilian regions but we could also predict the origin of the samples inside R1b1a-M269 to investigate the differences in the European background of these regions.

## Material and Methods

### Ethics Statement

Samples involved in this study are long-lasting anonymized DNA extracts previously obtained with informed written consent from healthy individuals for research purposes.

This work follows the ethical principles stated in the Helsinki Declaration (2000) of the World Medical Association, and it was approved by the institutional review board of the Laboratório de Genética Humana e Médica, Instituto de Ciências Biológicas, Universidade Federeal do Pará.

### Population Sample and DNA Extraction

A total of 1217 non-related male samples were collected to represent the five geopolitical regions of Brazil (see S1 Fig for sample locations and sizes). These samples are a randomly selected subset of those previously investigated by Palha et al. [10] for 23 Y chromosome STRs; and DNA extraction and quantification were performed as described therein.

### Genotyping methods

In this work, 41 Y-SNPs were analysed. These markers were selected based on the Y chromosome phylogeny [20] in order to resolve the haplogroups that are usually found in South American admixed populations, including the most common sub-Saharan African, Native American and European haplogroups (see S2 Fig with a phylogenetic tree including the selected markers).

SNP typing was performed through multiplex PCR and Single Base Extension (SBE) analysis using the SNaPshot kit (Thermo Fisher Scientific Inc.). To avoid genotyping all SNPs in each sample, a hierarchical approach based on the phylogeny reported by Van Oven et al. [20] was used to select the SNPs needed to define each haplogroup.

We first performed an initial screening of the major clades present in our population sample by typing all samples using the Multiplex Major South American previously described by Geppert et al. [21].

Subsequently we performed four specific multiplexes according to the previous results: (a) samples with the derived allele at M213 were further genotyped for the markers included in the Multiplex GIJ [22]; (b) samples with M207 derived allele were genotyped for Multiplex R that was designed and standardized in this work (see S1 Table for information on Multiplex R: markers included, primer sequences and PCR and SBE reaction conditions); (c) samples with derived allele at P170 were genotyped for Multiplex E [23] and; (d) finally, samples with the derived allele at M242 were genotyped for Multiplex Q [24].

### Statistical Analysis

Haplogroup frequencies were determined by direct counting. Population comparisons and genetic diversities estimated according to Nei [25] were performed with the software Arlequin 3.5.1.2 [26].

Population pairwise genetic distances were conducted based on *F*_ST_ and the significance was tested with 10,000 permutations. Pairwise genetic distances were visualized in two dimensional space using the multi-dimensional scaling (MDS) analysis included in the StatSoft, Inc. (2007) program, STATISTICA (data analysis software system), ver.8.0 (www.statsoft.com). This software was also used in Principal Component Anaysis (PCA).

Migration rates for the Brazilian populations were estimated using ADMIX 2 [27]. This software takes into account molecular information from any number of parental populations to compute the admixture coefficient (mγ) described by Bertorelle and Excoffier [28]. The computation of mγ was done taking into account distances between haplogroups, estimated as the differences in the number of substitution, and a specific Y chromosome mutation rate of 8.71 x 10^−10^ [29]. All the runs of ADMIX 2.0 were carried out fitting 1,000 random bootstrap samples.

A total of 382 out of the 630 samples belonging to haplogroup R-M207 were genotyped for the downstream SNPs M269, L23, U106 (S21), S116 (P312), U152 (S28), M529 (S145), M153 and M167 (SRY2627).

The frequency of each sub-haplogroup detected inside haplogroup R1b1a-M269 were compared with those found in Portugal, Spain, France, Italy, Germany, the Netherland and Turkey. The sub-haplogroup frequencies for the European populations were calculated using published data by Myres et al. [30] except for the Portuguese sample that was extracted from Busby et al. [31]. Therefore, the following samples have been pooled: Cantabria, Santander, Castille and Leon (representing Spain); Central Portugal, North Portugal and South Portugal (representing Portugal); France East, France, France West and France South (representing France); Germany East, Germany, Germany North, Germany South and Germany West (representing Germany); Italy, Italy North and Italy South (representing Italy); Turkey and Turkey, Cappadocia (representing Turkey).

## Results and Discussion

### Y chromosome haplogroups in Brazil

A total of 22 different haplogroups were detected in the whole sample, revealing the three major continental origins of the current Brazilian population, namely from America, Europe and Africa (Table 1).

**Table 1:** Continental origin of each haplogroup detected in the present study and haplogroup frequency distribution and haplotype diversity (HD) in the whole sample from Brazil as well as in the 5 studied samples from each geopolitical region.

Haplogroup	Origin	Brazil	North	Northeast	Central-West	Southeast	South
E1a- M33	Africa	0.002	0.006	0.006	-	-	-
E1b1a-M2	Africa	0.049	0.044	0.082	0.021	0.074	0.013
E1b1a-M191	Africa	0.026	0.017	0.041	0.010	0.032	0.022
E1b1a-M154	Africa	0.001	-	-	-	-	0.004
E1b1b-M35	Africa	0.004	-	0.012	-	0.005	-
E1b1b-M78	Africa/Europe	0.060	0.039	0.064	0.041	0.074	0.065
E1b1b-M81	Europe	0.031	0.022	0.023	0.021	0.043	0.035
E1b1b-M123	Europe	0.014	0.017	0.012	0.041	0.011	0.004
G-M201	Europe	0.051	0.054	0.074	0.031	0.027	0.069
I-M170	Europe	0.089	0.100	0.115	0.061	0.082	0.077
J-P209	Europe	0.101	0.096	0.103	0.160	0.094	0.082
KLT-M9	Europe	0.022	0.026	0.012	0.037	0.021	0.021
Q1a2-M346	America	0.001	-	-	-	-	0.004
Q1a2-M3	America	0.030	0.081	0.012	0.015	0.018	0.017
R-M207*	Europe	0.019	0.005	0.011	0.022	0.010	0.055
R1b1a-L23*	Europe	0.019	0.010	0.033	0.065	0.005	0.014
R1b1a-U106	Europe	0.043	0.031	0.022	0.033	0.039	0.090
R1b1a-S116*	Europe	0.325	0.342	0.314	0.327	0.332	0.317
R1b1a-U152	Europe	0.057	0.056	0.043	0.055	0.059	0.069
R1b1a-M529	Europe	0.043	0.046	0.022	0.065	0.054	0.028
R1b1a-M153	Europe	0.001	-	-	-	0.005	-
R1b1a-M167	Europe	0.009	0.010	-	-	0.015	0.014
HD ± s.e.		0.856±0.007	0.848±0.016	0.859±0.015	0.849±0.023	0.853±0.015	0.860±0.016

More than 50% of the Y chromosomes belong to the R1 branch, namely to the sub-lineage R1b1a-M269, which presents high frequencies throughout Western Europe. Previous studies have shown that Y-STR haplotypes are rather uninformative towards detecting R1b1a-M269 sub-haplogroups (and geographic origins), due to a recent expansion of this haplogroup in Europe, in a relatively short time period [32,33]. However, the R1b1a-M269 lineage can be further subdivided into several sub-lineages using Y-SNPs. These sub-lineages have high phylogeographic specificity and their frequencies are not homogeneously distributed on the European continent [30,31].

Haplogroup R1b1a-S116*, which has its greatest frequency in Iberia was, by far, the most frequent haplogroup observed in our sample, representing 32.5% of the Y chromosomes investigated [34,35]. Other R1b1a-M269 sub-lineages, more prevalent in other parts of Europe were also detected, including R1b1a-L23*, R1b1a-U106, R1b1a-U152 and R1b1a-M529 [31,34].

Other typical European haplogroups were also observed, namely J-P209, I-M170, G-M201, KLT-M9 and R-M207*. When adjusted to the total number of European lineages, these haplogroup frequencies are in the same range of those usually observed in Iberian populations [35].

Haplogroup E-P170 can be observed in Africa, Europe and the Middle East. Inside haplogroup E, some lineages originating in sub-Saharan Africa were detected in Brazil, namely E1a-M33, E1b1a-M2 and its M191 sub-lineage, E1b1a-M154 and E1b1b-M35, accounting for 8.2% of all Y chromosomes investigated here. E1b1b-M78 can be found at similar frequencies in Europe and Africa; hence, samples from this haplogroup were not included when estimating ancestry proportions. E1b1b-M81 exhibits a high prevalence in North Africa and Iberia, the latter being the probable origin of most Y chromosomes in Brazil belonging to this haplogroup. A low frequency of E1b1b-M123 was found in our sample, a haplogroup that is spread all over Europe and also frequent in some West Asian countries such as Turkey, Syria and Lebanon [36].

The haplogroup Q1a2-M346 and its sub-lineages, mainly Q1a2-M3, are almost completely restricted to Native American populations [37]. These lineages are usually poorly represented in current Brazilian admixed populations [11–15] and they were detected in only 3.1% of the Y chromosomes included in the present study.

**Fig 1:**
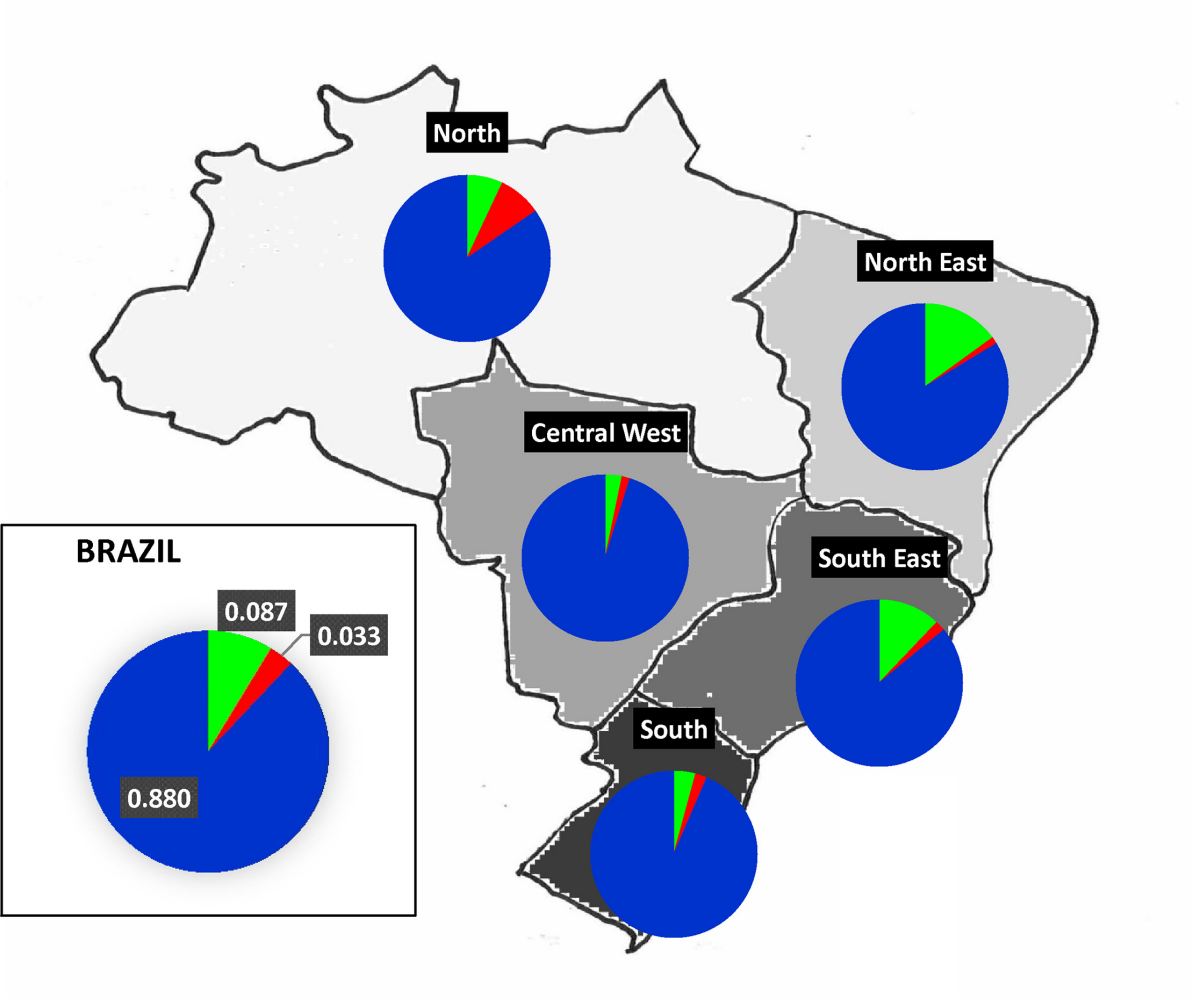
European (blue), African (green) and Native American (red) Y chromosome ancestry estimates in Brazilian admixed populations, obtained after adding all lineages from the same continental source and exclude those of unknown origin (which is the case of haplogroup E1b1b-M78).

Only very few and isolated populations in Brazil did not receive European admixture, either through early contact with the Portuguese or through more recent contact with other Europeans or European descendants in Brazil [16–19]. As expected from historical data and previously published works concerning the paternal ancestry of the Brazilian admixed populations [11–19], European lineages were the most frequent in the studied sample followed by African lineages, and Native American lineages were the least represented (Fig 1).

### Comparison of the different geopolitical regions in Brazil

Table 1 lists all haplogroups detected in each geopolitical region of the country, and the corresponding frequencies.

Haplogroup diversity remained constant among the five geopolitical regions of the country (Table 1), with a mean value of 0.856±0.007. This value can be considered high when compared with that previously reported for the Rio de Janeiro population (0.7589) [11]. This is explained by the high diversity of European lineages in Brazilian admixed populations that was not investigated before. Indeed, when haplotype diversities were re-calculated pooling the R1b1a-M269 sub-lineages (not discriminated before in the sample from Rio de Janeiro), significantly lower values of haplogroup diversity were observed in the samples from the South (0.6316±0.0341) and Central-West (0.6556±0.0412) regions when compared with Rio de Janeiro. The Northeast region presents the highest value of diversity (0.7642±0.0235) followed by the North (0.7227±0.0261) and the Southeast (0.7029±0.0251). These results are in agreement with the different admixture levels in the five Brazilian regions, with higher diversity in those with lower European contribution.

**Fig 2:**
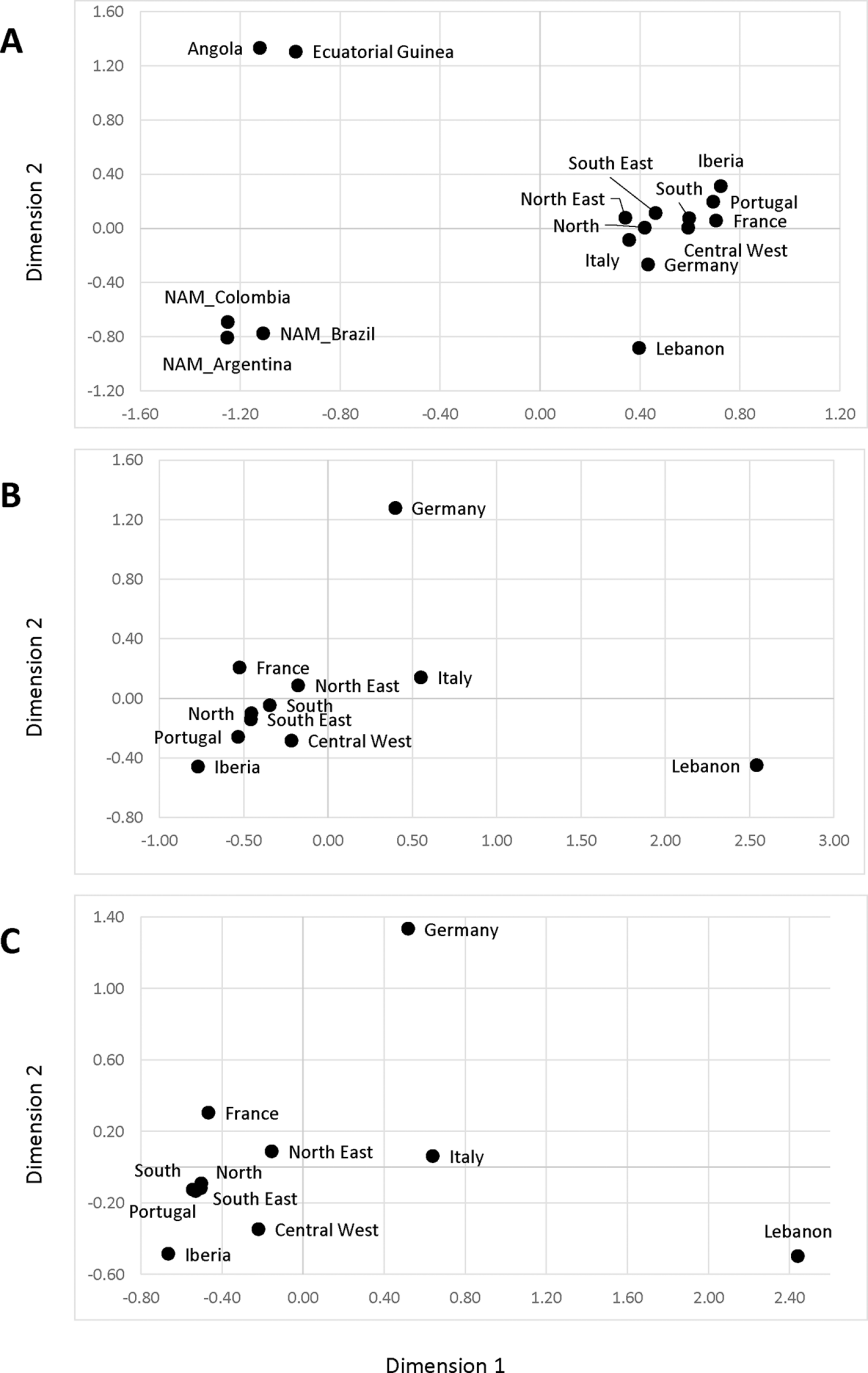
Multidimensional scaling plot of the pairwise F_ST_ genetic distances between: (A) Y-haplogroup frequencies found in Brazilian, Native American [38–40] European [41–46] and African [47–48] population samples (stress = 0.08148); (B) after excluding Sub-Saharan and Native American population samples (stress = 0.01356); (C) and after excluding Sub-Saharan and Native American Y chromosome lineages (stress = 0.00600).

Each geopolitical region of the country has its own history of colonization and settlement, and therefore, a genetic heterogeneity could be expected among their paternal lineages [1,5,10]. Population comparisons were performed among samples from different regions of Brazil, as well as between the Brazilian, Native American [38–40], European [41–46] and African [47,48] population samples. F_ST_ genetic distance did not reveal statistically significant differences in haplogroup frequency distributions among populations from different regions in Brazil (S2 Table). When comparing the Y-haplogroup frequencies in Brazil with those in other populations, the lowest distances were obtained with the Europeans, in particular Western European populations. However, both Northeastern and Southeastern regions exhibited significant differences with Portugal and France while exhibiting lower distances to sub-Saharan African populations than the samples from other regions. Large, statistically significant genetic distances were observed between all Brazilians and the African and Native American populations (S2 Table; Fig 2A). In the MDS plot of F_ST_ between Brazilian, European and Lebanese samples (Fig 2B), the samples from the South and Central-West are close to Portugal, the North stands between Portugal, France and Italy, and the Northeastern and Southeastern populations show a slight deviation toward the Middle Eastern Lebanon sample.

### The European genepool of the different geopolitical regions in Brazil

Genetic distances between the Brazilian, European and Lebanese samples were again calculated for Y-haplogroup frequencies after removing the sub-Saharan and Native American haplogroups. When considering only European ancestry, the samples from the South, the North and the Southeast were very close to Portugal (Fig 2C). The Northeast and Central-West samples are slightly displaced towards central European and Lebanese populations, respectively.

To predict the intracontinental European contributions to the five geopolitical regions in Brazil, migration rates were calculated using ADMIX 2.0 [27], considering Portugal, France, Italy, Germany and Lebanon as the parental populations. In this analysis, Sub-Saharan and Native American Y chromosome lineages were excluded and the frequencies of the remaining haplogroups were proportionally adjusted to a sum of 1.

The results showed differences among regions (Table 2). Portugal was estimated to be the main source of the male European lineages to Central-West, Southeast and South Brazil. The North and the Northeast showed the highest contribution from France and Italy, respectively. The highest migration rate from Lebanon was to the Central-Weast, whereas a significant migration from Germany was observed to the Central East, Southeast and South.

**Table 2:** Admixture coefficients (mγ ± SD) in the five Brazilian regions and corresponding normalized values (N-mγ), estimated using the ADMIX 2.0. Portugal [41], France [44], Italy [43], Germany [45] and Lebanon [46] were used as parental populations.

	North		Northeast		Central-West		Southeast		South		
	Mγ	N-mγ	Mγ	N-mγ	mγ	N-mγ	Mγ	N-mγ	mγ	N-mγ	
Portugal	0.45±0.36	**0.36**	0.17±0.52	**0.18**	1.20±0.47	**0.45**	1.24±0.46	**0.42**	0.87±0.39		**0.63**
France	0.71±0.44	**0.52**	0.08±0.62	0.14	0.08±0.72	0.17	-0.75±0.64	0.00	-0.11±0.59		0.00
Italy	-0.10±0.53	0.01	1.04±0.96	**0.61**	-0.61±0.75	0.00	0.52±0.66	**0.27**	0.11±0.67		0.14
Germany	-0.12±0.10	0.00	-0.07±0.14	0.07	0.02±0.16	0.16	0.14±0.16	0.19	0.16±0.14		**0.17**
Lebanon	0.07±0.16	0.12	-0.21±0.30	0.00	0.31±0.24	**0.23**	-0.14±0.20	0.13	-0.04±0.21		0.04

**Note**: The highest contributions to each region are in bold

### Native American, African and European contributions

Haplogroup Q1a2-M3 was found with a high prevalence in the North region, whereas in other regions the frequency of this haplogroup was not higher than 2.1% (Fig 1). A similarly high frequency of Native American Y-haplogroups was previously found in a population sample from Manaus, also in the North region [15]; low frequencies have also been reported in populations from the remaining regions [11–14].

These results are consistent with several studies using various types of genetic markers, showing a higher Native American ancestry in populations from the North when compared with other regions of the country [6–9].

The highest African contribution was observed in the Northeast, followed by the Southeast, and much lower frequencies were observed in the remaining regions (Fig 1). The total frequency of African haplogroups (E1a-M33, E1b1a-M2, E1b1a-M191, E1b1a-M154 and E1b1b-M35) was 14.1% in the Northeast and 11.1% in the Southeast. When adjusting these values by accounting for the percentage of haplogroups with unknown ancestry, African ancestry was estimated to be 14.9% in the Northeast and 12.1% in the Southeast. Although there are no previous estimates for the African contribution to these two regions using a similar set of Y-SNP markers, a recent study reported a high proportion of African male lineages in Rio de Janeiro (15.73%), in the Southeast [11]. While sample sizes can justify the observed difference, the population substructure cannot be ruled out because the samples included in the present study belong to two other districts (São Paulo and Minas Gerais), excluding Rio de Janeiro.

The two remaining populations from the Central-West and the South are those with the highest number of European haplogroups (91.3% and 87.5%, respectively). The estimates for the European male contributions are 95.2% and 93.6% for the Central-West and the South, respectively. A clear difference emerges when examining the distribution of European haplogroups in these two samples, with haplogroup E1b1b-M123 ten times more frequent in the Central West than in the South; and haplogroup J-P209 is also two times more frequent in the Central-West sample. Moreover, haplogroups E1b1b-M123 and J-P209 present higher frequencies in Central-Western Brazil (4.1% and 16.0%, respectively) than in Portugal (1.2% and 10.4%, respectively).

### Analysis of R1b1a-M269 subtypes

R1b1a-M269 is the most frequent haplogroup in Europe, presenting a cline distribution with high frequencies in the West, decreasing towards the East. The highest frequencies of this haplogroup were reported for populations in Ireland [49]. Its prevalence is also high across the Iberian Peninsula, especially in populations from Basque Country and the Pyrenees [27,50,51,52].

R1b1a-M269 is also present in significant proportions in South American populations as the most frequent haplogroup in the great majority of admixed, non-Native populations from Brazil, Colombia and Argentina [11,53,54].

In a survey of 75 Y chromosomes from African Americans and European Americans belonging to the haplogroup R1b1a-M269, Sims et al. [55] were able to further differentiate this lineage into 5 distinct sub-haplogroups by genotyping M222 and three other previously uncharacterized SNPs (U152, U106 and U198) downstream of M269. In this study, a correlation was found between the samples carrying the M222-derived allele and the Irish Modal Haplotype (IMH) described by Moore et al. [49].

Further studies using SNPs to increase the discrimination between lineages inside haplogroup R-M207 were performed in large samples from West Asian and European populations, revealing different gradients for R1b1a-M269 sub-clades inside Europe [30,31]. The L11-derived allele (also known as S127) separates Western European from the Eurasian lineages. The sub-haplogroup R1b1a-U106 (S21) is more frequent in Central and Eastern Europe, reaching 66.8% in Germany, while R1b1a-S116, more frequent in the Western portion of the continent, is further subdivided into several haplogroups. The sub-lineage R1b1a-S116* is the most frequent in the Iberian Peninsula, R1b1a-U152 is more frequent in France and Italy, and R1b1a-M529 has higher frequencies in England and Ireland. The sub-lineages R1b1a-M153 and R1b1a-M167 were described at high frequencies in Basque Country. R1b1a-M167 was also found at high frequencies in the Pyrenees [30,50,51]. The frequencies of the sub-haplogroups investigated inside R-M207 in each geopolitical region are indicated in S3 Table.

Concerning the European lineages, the haplogroup R1b1a-S116* was the most frequent in the five geopolitical regions of the country emphasizing the strong influence of the early Portuguese colonization [30,31].

To investigate possible signs of differential European colonization in the five regions of Brazil, the frequency distributions of the R1b1a-L23*, U106, S116*, U152, M529, M153 and M167 sub-haplogroups with respect to total R1b1a-M269 were compared by means of pairwise F_ST_ values calculated among Brazilian and some European populations representing potential sources of male immigrants to Brazil including Portugal, Spain, Italy, Germany and Netherlands. The sample from Turkey was also included to represent the immigration from the Middle East because no data are available for other countries in this region with reported historically affinities with Brazil (like Syria and Lebanon) [1].

**Fig 3:**
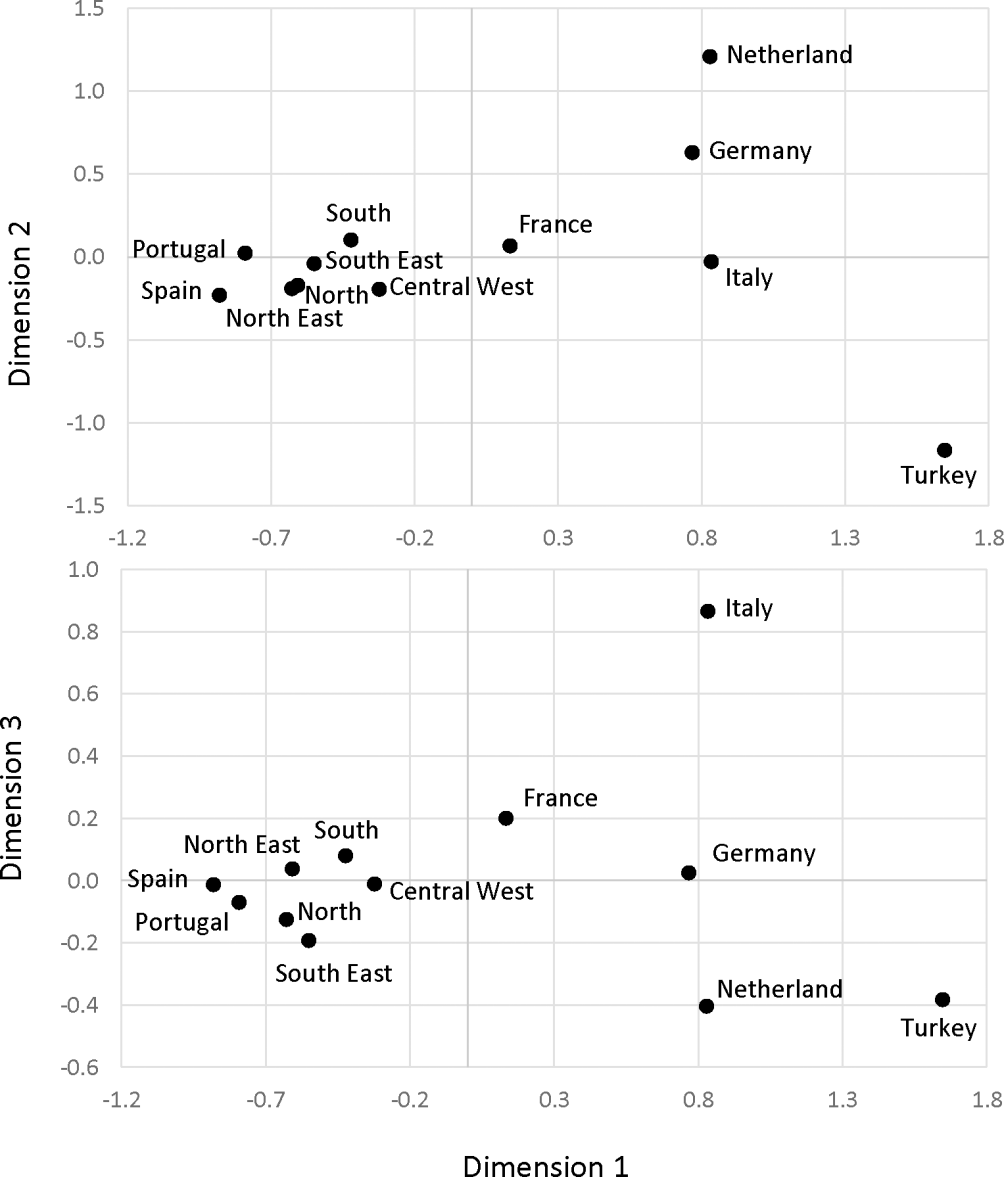
Multidimensional scaling plot of the pairwise *F*_*ST*_ genetic distances based of the frequencies of R-L23*, R-U106, R-S116*, R-U152 and R-M529 haplogroups in the five regions of Brazil (see S3 Table), and in samples from different European populations that potentially have contributed to the nowadays Brazilian Y chromosome gene pool. European data were extracted from Myres et al. [30] and Busby et al. [31]. Samples from the same country were pooled when no statistically significant differences between them were found (see Material and Methods for details). Stress = 0.0022310.

**Fig 4:**
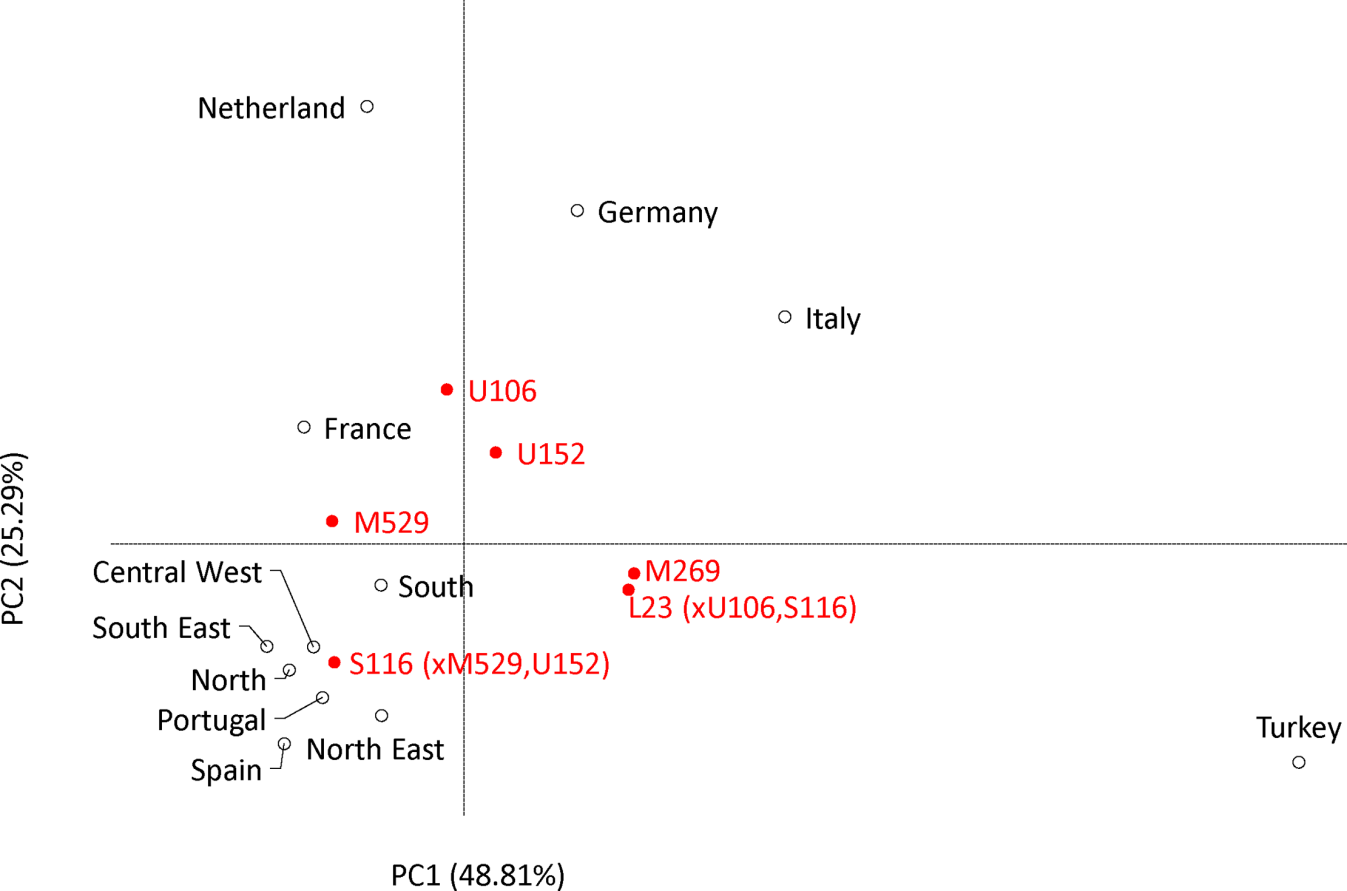
Principal component analysis of R-L23*, R-U106, R-S116*, R-U152 and R-M529 haplogroup frequencies in the five regions of Brazil (see S3 Table) and in samples from different European populations that potentially have contributed to the nowadays Brazilian Y chromosome gene pool. European data were extracted from Myres et al. [30] and Busby et al. [31]. Samples from the same country were pooled when no statistically significant differences between them were found (see Material and Methods for details).

The results showed no statistically significant differences among the Brazilian samples or between the Brazilian and Iberian populations (S4 Table). Except for the North and Southeast, no significant differences were detected in the comparison of the Brazilian and the French samples. High F_ST_ values were found between Brazil and the remaining populations, associated with very low values of non-differentiation probabilities. In the MDS plot of the pairwise genetic distances (Fig 3), the five Brazilian and Iberian samples clustered together. The samples from the South and Central-West are more distant from the Iberian samples in the first dimension; accounting for the second and third dimensions the South is less distant from Germany, and the Central-West is less distant from Turkey than the remaining samples from Brazil. The PC analysis (Fig 4) supported previous results showing a closer relationship between the Brazilian and Iberian populations. The first and second dimensions capture 48.81% and 25.29% of the total inertia, respectively. The first axis mainly separates the Western and Central European samples from Turkey, which is characterized by high frequencies of haplogroups R1b1a-M269 (xL23) and R1b1a-L23 (xU106, S116). The second axis separates Iberia with a high frequency of R-S116* from other European countries, for which position reflects a higher frequency of haplogroups R1b1a-M529, R1b1a-U106 and R1b1a-U152. The position of the samples from the North and the Southeast are compatible with a strong contribution of European male lineages from Iberia; the South shows signs of a non-negligible Central European influx (possibly from Germany and Italy); and the Northeast appears to have a higher Eastern European contribution than do other Brazilian regions.

The results of the admixture analysis for the R1b1a-M269 sub-haplogroups (Table 3), while reinforcing the main contribution of Iberia to all Brazilian populations, also emphasize the German contribution to the South.

**Table 3:** Admixture coefficients for the R1b1a-M269 sub-lineages in the five Brazilian regions and corresponding SD values, estimated using the ADMIX 2.0. Spain [30], Portugal [31], Netherlands [30], France [30], Germany [30], Italy [30] and Turkey [30] were used as parental populations.

	North		Northeast		Central-West		Southeast		South	
	mγ	N-mγ	mγ	N-mγ	mγ	N-mγ	mγ	N-mγ	mγ	N-mγ
Spain	0.47±0.73	**0.34**	0.79±0.89	**0.52**	0.08±1.15	0.11	0.36±0.84	**0.27**	0.69±0.75	**0.46**
Portugal	0.49±0.93	**0.35**	0.23±1.14	**0.20**	0.82±1.46	**0.47**	0.55±1.16	**0.37**	0.11±0.95	0.12
Netherlands	0.02±0.22	0.07	-0.05±0.22	0.03	0.07±0.27	0.11	0.05±0.23	0.09	0.01±0.18	0.07
France	0.17±0.29	0.16	-0.10±0.32	0.00	0.27±0.42	**0.21**	0.25±0.27	0.20	-0.10±0.27	0.00
Germany	-0.06±0.41	0.02	0.09±0.41	0.11	-0.15±0.53	0.00	-0.07±0.44	0.02	0.26±0.33	**0.21**
Italy	0.01±0.18	0.06	0.04±0.24	0.08	-0.08±0.29	0.03	-0.02±0.20	0.05	0.08±0.18	0.10
Turkey	-0.09±0.14	0.00	0.00±0.18	0.06	0.00±0.22	0.07	-0.11±0.19	0.00	-0.05±0.14	0.03

**Note**: The highest contributions to each region are in bold

### Conclusions

The set of polymorphisms selected were able to discriminate the origin of Y chromosome haplogroups in America, Africa and Europe in Brazilian admixed populations, thereby contributing to a better characterization of Brazilian paternal ancestry. Corroborating historical and previous genetic data, a high European ancestry was detected across the country. However, the differences observed among populations from the five geopolitical regions indicate a population stratification caused by a variation in Native American and African contributions, supporting different colonization/migration models [11–15]. The highest Native American ancestry (detected through the presence of haplogroups Q1a3*-M346 and Q1a3a1a*-M3) was found in the North, a sparsely populated region that includes most of the Amazonia territory, inhabited by a large number of Native American communities [7,8,15].

The largest proportions of sub-Saharan African Y chromosomes (represented by haplogroups E1b1a1*-M2 and E1b1a1f-M191) were found in the Eastern populations. Historically, the Eastern regions were important destinations of African slaves, which entered Brazil through the port cities of Salvador (in the northeast Atlantic coast), Rio de Janeiro and Santos (in the southeast Atlantic coast) [1–4].

A detailed analysis of the European Y chromosome gene pool also allowed for the detection of differences among Brazilian populations and the evaluation of the genetic impact of the early Portuguese colonization and the more recent migrations from other European countries. The Northeast region showed the largest genetic distance to Portugal and a slight increase in the frequency of haplogroup R1b1a-L23*, which can be explained by the Eastern European influx (also observed in the PCA).

Most of the Germans and Italians that arrived in Brazil during the nineteenth and early twentieth centuries settled in the South [1]. The frequency of haplogroups R1b1a-U106 and R1b1a-U152 increases in this region, indicating an influx of Y-lineages from Central Europe, namely from Germany (where haplogroup R1b1a-U106 has a high frequency) and Italy (where R1b1a-U152 has a high frequency).

The Central-Western region was the last to be settled in the country by migrants from other Brazilian regions, mainly the Northeast and Southeast. This situation attracted many Arab traders who arrived and settled. The high frequencies of haplogroups R1b1a-L23*, E1b1b-M123 and J-P209 found in this region can be explained by the influx from Near East, since these lineages are frequent there [30–36].

Population comparison based on pairwise F_ST_ genetic distances among populations from the different regions did not reveal statistically significant differences in haplogroup frequency distributions, which can be explained by the high predominance of Western European Y chromosomes in all populations. However, the close agreement between the genetic differences observed among the geopolitical region and the history of colonization and settlement of the country supports a possible population stratification of the paternal lineages in Brazil that needs to be further investigated in larger sample sets of each region.

## Supporting Information

S1 Fig Map of Brazil subdivided into five geopolitical regions, indicating sampling locations and sizes, for the 1217 samples included in this study. (PDF)

S2 Fig Phylogenetic tree of Y-haplogroups analyzed in the present study. The haplogroups are named in accordance with Van Oven et al. [20].
(PDF)

S1 Table PCR and Single Base Extension (SBE) primer sequences and reaction condition for Multiplex R. (PDF)

S2 Table Matrix showing the pairwise FSTs (below diagonal) among the 5 regions of Brazil, Portugal [41], Iberia [42], France [44], Italy [43], Germany [45], Lebanon [46], Angola [48], Equatorial Guinea [47] and Native Americans from Colombia [40], Brazil [39], and Argentina [38]; and the corresponding differentiation p values (above diagonal) obtained for 50,175 permutations (s.e.≤0.0022). Significant non-differentiation *p*-values are indicated in red; for a significance level of 0.00042, obtained by applying the Bonferroni correction for multiple tests.
(PDF)

S3 Table Frequency distribution of R1b1b-M269 sub-haplogroups with respect to total of samples from haplogroup R1b1b-M269 in the five geopolitical region of Brazil and in the other population samples used for comparison. (PDF)

S4 Table Matrix of the pairwise *F*_*ST*_ genetic distances among the 5 geopolitical regions of Brazil and seven European populations (below diagonal) and the corresponding differentiation *p* values (above diagonal) obtained for 10,100 permutations (s.e.≤0.0038). *F*_*ST*_ values were calculated based of the frequencies of R1b1a-L23*, R1b1a-U106, R1b1a-S116*, R1b1a-U152 and R1b1a-M529 haplogroups that are indicated in S3 Table.
(PDF)

S5 Table List of Y chromosome SNP haplogroups found in samples from the 5 geopolitical regions of Brazil (XLSX)
