# Supplementary material for: Male Lineages in Brazil: Intercontinental Admixture and Stratification of the European Background
Source: PLoS One. 2016 Apr 5;11(4):e0152573. doi: 10.1371/journal.pone.0152573 (PMC4821637; doi:10.1371/journal.pone.0152573)
Supplement: S1 Fig — (PDF) [file pone.0152573.s001.pdf]

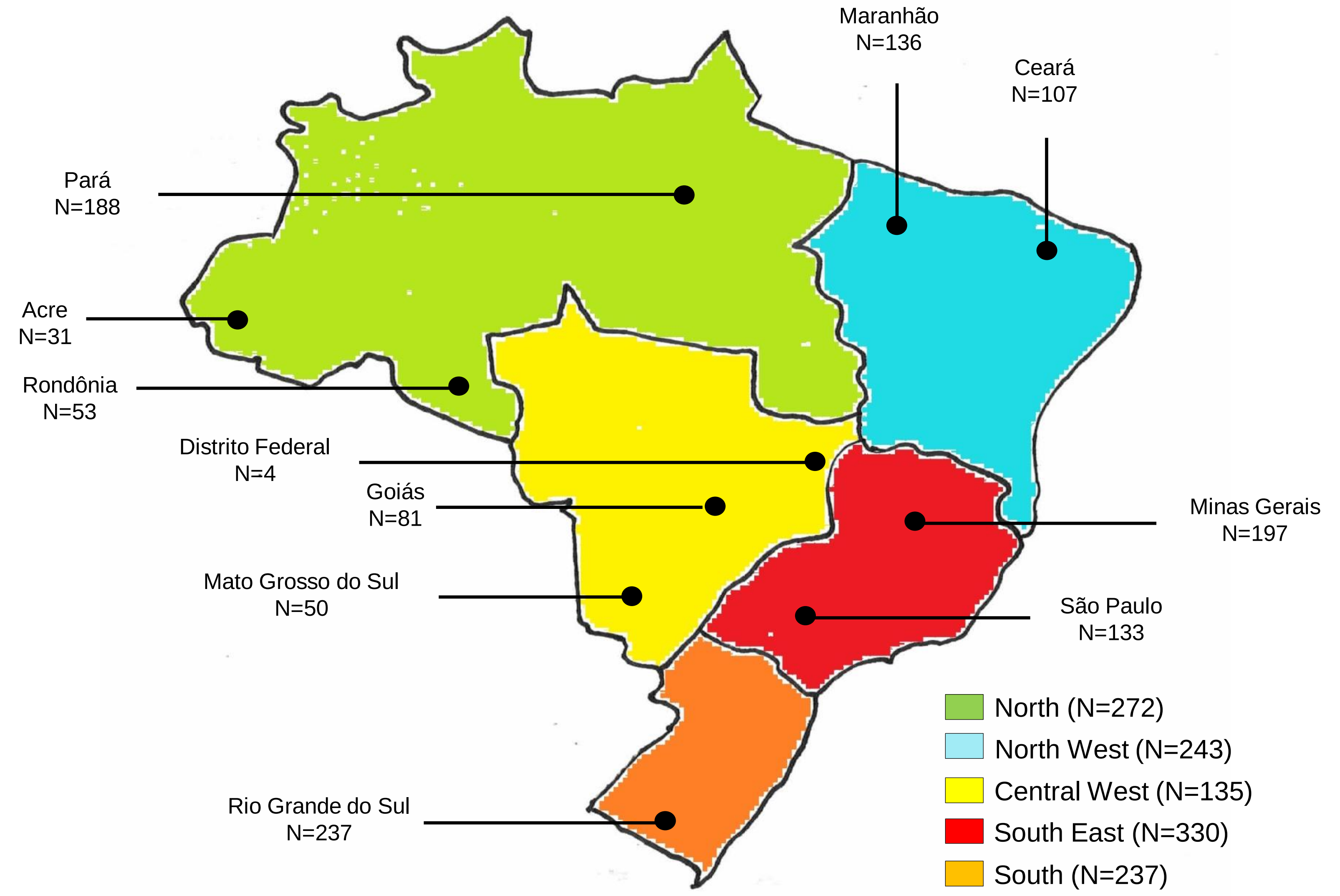

S1 Fig. Representation of Brazil subdivided into five geopolitical regions, indicating sampling locations and sizes, for the 1217 samples included in this study.
