# Supplementary material for: Male Lineages in Brazil: Intercontinental Admixture and Stratification of the European Background
Source: PLoS One. 2016 Apr 5;11(4):e0152573. doi: 10.1371/journal.pone.0152573 (PMC4821637; doi:10.1371/journal.pone.0152573)
Supplement: S2 Fig — The haplogroups are named in accordance with Van Oven et al. [20]. (PDF) [file pone.0152573.s002.pdf]

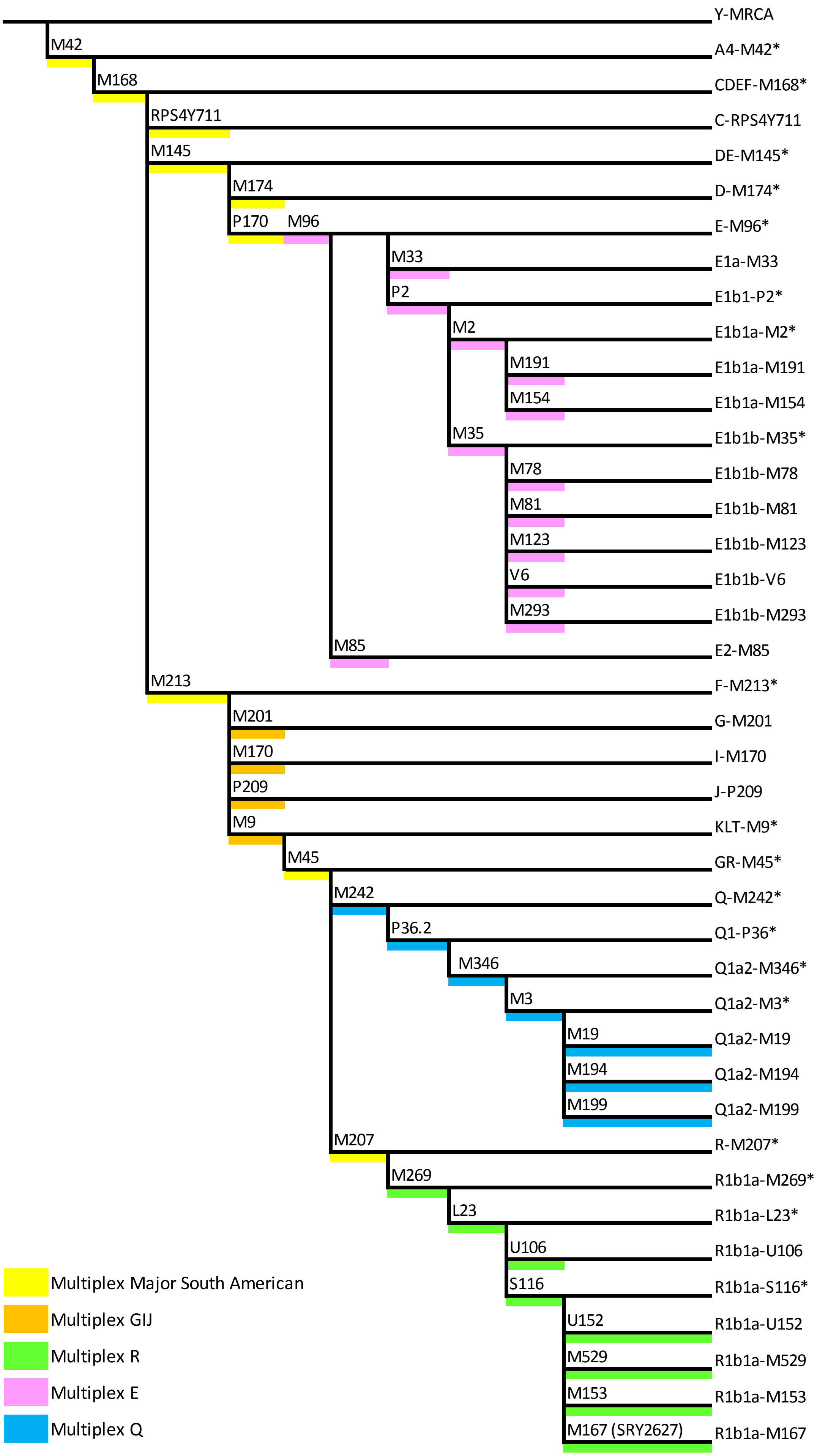

S2 Fig. Phylogenetic tree of Y-haplogroups analyzed in the present study. The haplogroups are named in accordance with Van Oven et al.[20]
