# Supplementary material for: Male Lineages in Brazil: Intercontinental Admixture and Stratification of the European Background
Source: PLoS One. 2016 Apr 5;11(4):e0152573. doi: 10.1371/journal.pone.0152573 (PMC4821637; doi:10.1371/journal.pone.0152573)
Supplement: S1 Table — (PDF) [file pone.0152573.s003.pdf]

S1 Table. PCR and Single Base Extension (SBE) primer sequences and reaction condition for Multiplex R.

**PCR***				
Marker	*SNP*	*PCR Forward Primer (5'-3')*	*PCR Reverse Primer (5'-3')*	*PCR fragment size (bp)*
M269	T>C	CATGCCTAGCCTCATTCCTC	CTGGATGGTCACGATCTCCT	249
L23	G>A	ACACAGTGAAACCCCGTCTC	AAGATTGTGGGGACAAAGGA	229
U106	C>T	TCCTGAATAGCAAATCCCAAAG	AATGGCAGAGGTAGGAGGAAAT	192
U152	G>A	GAAACATTCCACGCTTGAGG	AGCCTCTTTTTGGCTTCCAT	163
S116	C>A	TCAGTCAGGGCAAATCTGAA	GGTGGAGTTGGGGCTAAAGT	241
M529	C>G	GCCCCCAAAACAACAGAATA	GGAAGCATTCAGAAGCAGGT	228
M153	T>A	TTCTCAGACACCAATGGTCCTA	TCTGACTTGGAAAGGGGAAA	76
M167	C>T	GAGGCTGGGCCAAGTTAAG	CTTCCTCGGAACCACTACCA	131
M207	A>G	CGTTACAACTATGGGGCAAA	TCCTCTCTGAAATGCCGAAT	322
**SNaPshot****				
Y-SNP	***	*SBE Primer (5'-3')*	*Concentration in the reaction (µM)*	*SBE fragment size (bp)*
M269	*For*	GTCTGACAAGGAATGATCAGGGTTTGGTTAAT	0.12	32
L23	*For*	GCGACAGAGCGAGACTCT	0.16	18
U106	*Rev*	TCTGACAATAGCAAATCCCAAAGCTCCA	0.12	28
U152	*Rev*	CAAGGATAAGAAAAATGAGTATTGTGAAAATA	0.08	32
S116	*Rev*	GAAAGTCTGACAAGAGTTGGGGCTAAAGTGAAAG	0.04	35
M529	*For*	AATAACAACCGCTCTCTCAGACA	0.04	23
M153	*Rev*	AAAGCTCAAAGGGTATGTGAACA	0.04	23
M167 (SRY2627)	*For*	AAGCCCCACAGGGTGC	0.04	16
M207	*For*	AACAAATGTAAGTCAAGCAAGAAATTTA	0.10	28

*PCR was carried out in 10 µl containing: 5µl Qiagen® multiplex PCR kit, 0.2µM of each primer and 5-10 ng DNA. Thermocycling conditions: denaturation at 95ºC for 15 min; 35 cycles at 94ºC for 30s, 60ºC for 90s and 72ºC for 60s; final extension of 72ºC, 10 min. Prior to SNaPshot reaction, 1 µl of the PCR product was purified with 0.5 µl of Exo-SAP-it (USB), incubated at 37°C for 15 min, and followed by an enzyme inactivation at 85°C for 15 min. **SNaPshot was carried out in 5 µl reaction containing 1 µl of SNaPshot™ Multiplex Ready Mix (Applied Biosystems), 1.5 µl of purified PCR product and extension primers. The reactions were submitted to 25 cycles of 96ºC for 10 s, 50ºC for 5 s and 60ºC for 30 s. Final products were purified with 1 µl of SAP (USB) at 37ºC for 1 h, followed by a denaturing step of 85ºC for 15 min. The single-base extension products were run on an ABI 3130 Genetic Analyzer (Applied Biosystems), and the analysis was performed with GeneMapper® Software v4.0. ***Annealing of the SBE primers in forward or reverse sequence direction.
