# Supplementary material for: Male Lineages in Brazil: Intercontinental Admixture and Stratification of the European Background
Source: PLoS One. 2016 Apr 5;11(4):e0152573. doi: 10.1371/journal.pone.0152573 (PMC4821637; doi:10.1371/journal.pone.0152573)
Supplement: S2 Table — Significant non-differentiation p-values are indicated in red; for a significance level of 0.00042, obtained by applying the Bonferroni correction for multiple tests. (PDF) [file pone.0152573.s004.pdf]

S2 Table. Matrix showing the pairwise F_{ST}s (below diagonal) among the 5 regions of Brazil, Portugal [41], Iberia [42], France [44], Italy [43], Germany [45], Lebanon [46], Angola [48], Equatorial Guinea [47] and Native Americans from Colombia [40], Brazil [39], and Argentina [38]; and the corresponding differentiation *p* values (above diagonal) obtained for 50,175 permutations (s.e.≤0.0022). Significant non-differentiation *p*-values are indicated in red; for a significance level of 0.00042, obtained by applying the Bonferroni correction for multiple tests.

	Brazil_N	Brazil_NE	Brazil_CW	Brazil_SE	Brazil_S	Portugal	Iberia	France	Italy	Germany	Lebanon	Angola	Ecuatorial Guinea	NAM_Colombia	NAM_Brazil	NAM_Argentina
Brazil_N	*	0.0354	0.0731	0.0732	0.0650	0.0003	<5E-6	0.0003	<5E-6	<5E-6	<5E-6	<5E-6	<5E-6	<5E-6	<5E-6	<5E-6
Brazil_NE	0.0057	*	0.0054	0.0980	0.0026	<5E-6	<5E-6	<5E-6	0.00002	<5E-6	<5E-6	<5E-6	<5E-6	<5E-6	<5E-6	<5E-6
Brazil_CW	0.0056	0.0158	*	0.0635	0.1549	0.1052	0.0045	0.0089	0.0001	<5E-6	<5E-6	<5E-6	<5E-6	<5E-6	<5E-6	<5E-6
Brazil_SE	0.0034	0.0030	0.0058	*	0.0718	0.0002	<5E-6	0.00002	<5E-6	<5E-6	<5E-6	<5E-6	<5E-6	<5E-6	<5E-6	<5E-6
Brazil_S	0.0042	0.0134	0.0031	0.0037	*	0.2013	0.0028	0.0126	<5E-6	<5E-6	<5E-6	<5E-6	<5E-6	<5E-6	<5E-6	<5E-6
Portugal	0.0136	0.0290	0.0037	0.0134	0.0011	*	0.0167	0.0002	<5E-6	<5E-6	<5E-6	<5E-6	<5E-6	<5E-6	<5E-6	<5E-6
Iberia	0.0288	0.0519	0.0137	0.0268	0.0091	0.0027	*	<5E-6	<5E-6	<5E-6	<5E-6	<5E-6	<5E-6	<5E-6	<5E-6	<5E-6
France	0.0138	0.0251	0.0128	0.0183	0.0071	0.0088	0.0178	*	<5E-6	<5E-6	<5E-6	<5E-6	<5E-6	<5E-6	<5E-6	<5E-6
Italy	0.0227	0.0158	0.0237	0.0279	0.0274	0.0404	0.0677	0.0358	*	<5E-6	<5E-6	<5E-6	<5E-6	<5E-6	<5E-6	<5E-6
Germany	0.0655	0.0595	0.0797	0.0710	0.0668	0.0873	0.1196	0.0639	0.0500	*	<5E-6	<5E-6	<5E-6	<5E-6	<5E-6	<5E-6
Lebanon	0.1864	0.1636	0.1800	0.1932	0.2111	0.2305	0.2791	0.2280	0.1090	0.1863	*	<5E-6	<5E-6	<5E-6	<5E-6	<5E-6
Angola	0.3527	0.2996	0.4126	0.3334	0.3958	0.4226	0.4631	0.4159	0.3289	0.3616	0.3691	*	0.0887	<5E-6	<5E-6	<5E-6
Ecuatorial Guinea	0.3088	0.2551	0.3610	0.2901	0.3478	0.3807	0.4238	0.3746	0.2851	0.3089	0.3266	0.0121	*	<5E-6	<5E-6	<5E-6
NAM_Colombia	0.2475	0.2586	0.3096	0.2838	0.3060	0.3389	0.3855	0.3325	0.2443	0.2778	0.2887	0.4903	0.4285	*	0.0862	0.0524
NAM_Brazil	0.2287	0.2435	0.2946	0.2658	0.2911	0.3228	0.3674	0.3179	0.2389	0.2725	0.2969	0.4979	0.4258	0.0180	*	0.6035
NAM_Argentina	0.2578	0.2773	0.3248	0.2975	0.3192	0.3466	0.3902	0.3429	0.2617	0.2974	0.3148	0.5565	0.4814	0.0242	-0.0082	*
