# Supplementary material for: Male Lineages in Brazil: Intercontinental Admixture and Stratification of the European Background
Source: PLoS One. 2016 Apr 5;11(4):e0152573. doi: 10.1371/journal.pone.0152573 (PMC4821637; doi:10.1371/journal.pone.0152573)
Supplement: S3 Table — (PDF) [file pone.0152573.s005.pdf]

S3 Table. Frequency distribution of R1b1b-M269 sub-haplogroups with respect to total of samples from haplogroup R1b1b-M269 in the five geopolitical region of Brazil and in the other population samples used for comparison.

Haplogroup	North (n=97)	North East (n=41)	Central West (n=50)	South East (n=104)	South (n=77)	Spain (n=111)	Portugal (n=182)	Netherlands (n=46)	France (n=53)	Germany (n=141)	Italy (n=105)	Turkey (n=91)
R1b1a-M269*	0.0000	0.0000	0.0000	0.0000	0.0000	0.0000	0.0000	0.0000	0.0000	0.0213	0.0286	0.1319
R1b1a-L23*	0.0206	0.0732	0.1200	0.0096	0.0260	0.0270	0.1593	0.0000	0.0566	0.0638	0.1905	0.7912
R1b1a-U106	0.0619	0.0490	0.0600	0.0769	0.1688	0.0450	0.0659	0.6957	0.1321	0.4752	0.0952	0.0220
R1b1a-S116*	0.6907	0.7070	0.6000	0.6538	0.5974	0.7928	0.5824	0.1304	0.3962	0.1560	0.1333	0.0220
R1b1a-U152	0.1134	0.0980	0.1000	0.1154	0.1299	0.0541	0.1099	0.0652	0.2642	0.2340	0.5429	0.0330
R1b1a-M529	0.0928	0.0490	0.1200	0.1058	0.0519	0.0811	0.0824	0.1087	0.1509	0.0496	0.0095	0.0000
R1b1a-M153	0.0000	0.0000	0.0000	0.0096	0.0000	n.d.	n.d.	n.d.	n.d.	n.d.	n.d.	n.d.
R1b1a-M167	0.0206	0.0240	0.0000	0.0288	0.0260	n.d.	n.d.	n.d.	n.d.	n.d.	n.d.	n.d.

Note: The Y-SNPs M153 and M167 were only investigated in the Brazilian samples from this work and, therefore, this sub-haplogroups were pooled with R1b1a-S116* in comparisons with other populations. Not determined: n.d.
