# Supplementary material for: Male Lineages in Brazil: Intercontinental Admixture and Stratification of the European Background
Source: PLoS One. 2016 Apr 5;11(4):e0152573. doi: 10.1371/journal.pone.0152573 (PMC4821637; doi:10.1371/journal.pone.0152573)
Supplement: S4 Table — FST values were calculated based of the frequencies of R1b1a-L23*, R1b1a-U106, R1b1a-S116*, R1b1a-U152 and R1b1a-M529 haplogroups that are indicated in S3 Table. (PDF) [file pone.0152573.s006.pdf]

S4 Table. Matrix of the pairwise F_{ST} genetic distances among the 5 geopolitical regions of Brazil and seven European populations (below diagonal) and the corresponding differentiation *p* values (above diagonal) obtained for 10,100 permutations (s.e.≤0.0038). F_{ST} values were calculated based of the frequencies of R1b1a-L23*, R1b1a-U106, R1b1a-S116*, R1b1a-U152 and R1b1a-M529 haplogroups that are indicated in S3 Table.

	Brazil_North	Brazil_North East	Brazil_Central West	Brazil_South East	Brazil_South	Spain	Portugal	Netherlands	France	Germany	Italy	Turkey
Brazil_North	*	0.8303	0.20606	0.97393	0.15003	0.24672	0.01139	**< 5E-06**	**0.00020**	**< 5E-06**	**< 5E-06**	**< 5E-06**
Brazil_North East	-0.01218	*	0.43500	0.63023	0.22654	0.49024	0.17105	**< 5E-06**	0.00190	**< 5E-06**	**< 5E-06**	**< 5E-06**
Brazil_Central West	0.00713	-0.00175	*	0.23683	0.23068	0.01403	0.90930	**< 5E-06**	0.02947	**< 5E-06**	**< 5E-06**	**< 5E-06**
Brazil_South East	-0.00913	-0.0077	0.00531	*	0.23323	0.11332	0.00969	**< 5E-06**	**0.00055**	**< 5E-06**	**< 5E-06**	**< 5E-06**
Brazil_South	0.00877	0.00691	0.00631	0.00441	*	0.00425	0.03995	**< 5E-06**	0.01154	**< 5E-06**	**< 5E-06**	**< 5E-06**
Spain	0.00315	-0.00418	0.04040	0.00911	0.04360	*	**< 5E-06**	**< 5E-06**	**< 5E-06**	**< 5E-06**	**< 5E-06**	**< 5E-06**
Portugal	0.02333	0.00916	-0.01008	0.02269	0.01654	0.05264	*	**< 5E-06**	0.00300	**< 5E-06**	**< 5E-06**	**< 5E-06**
Netherlands	0.43101	0.44098	0.35764	0.40938	0.32000	0.51517	0.34132	*	**< 5E-06**	**< 5E-06**	**< 5E-06**	**< 5E-06**
France	0.09193	0.09091	0.03707	0.08001	0.04566	0.17183	0.04619	0.24495	*	**< 5E-06**	**< 5E-06**	**< 5E-06**
Germany	0.28764	0.28079	0.21957	0.27266	0.19359	0.36031	0.22041	0.04955	0.10664	*	**< 5E-06**	**< 5E-06**
Italy	0.32587	0.31248	0.24590	0.31570	0.25998	0.41119	0.23609	0.33912	0.10977	0.15480	*	**< 5E-06**
Turkey	0.56708	0.56575	0.47485	0.55816	0.52020	0.62374	0.40992	0.57869	0.43540	0.41496	0.38356	*

Note: in bold are indicated the *p*-values that are below the significance value 0.00076, obtained after applying Bonferroni's correction for multiple tests.
